# Leukocytoclastic Vasculitis as a Complication of Recombinant Granulocyte Colony-Stimulating Factor Therapy in a Heart Transplant Patient

**DOI:** 10.1155/2014/160407

**Published:** 2014-01-30

**Authors:** Giovanbattista Ippoliti, Marco Paulli, Marco Lucioni, Marinella Lauriola, Andrea Maria D'Armini

**Affiliations:** ^1^U.O Medicina Interna, Policlinico di Monza, Via C. Amati 111, 20900 Monza, Italy; ^2^Division of Cardiac Surgery, University of Pavia School of Medicine, Foundation “I.R.C.C.S. San Matteo” Hospital, Pavia, Italy; ^3^Anatomic Pathology, Foundation IRCCS Policlinico San Matteo, University of Pavia, Italy

## Abstract

Recombinant granulocyte colony-stimulating factor (rG-CSF) is a myeloid growth factor that is widely used in haematology to recover neutropenia secondary to myelosuppressive chemotherapy. Leukocytoclastic vasculitis is an acknowledged side effect of the above therapy. Its pathogenesis involves many mechanisms that collectively induce an increase in neutrophil function and a subsequent release of cytokines. Here, we report a case of leukocytoclastic vasculitis proven by skin biopsy, following the use of rG-CSF in a heart transplant patient with leukopenia secondary to immunosuppressive therapy.

## 1. Introduction

Vasculitis is an inflammation of the blood vessel wall that leads to various clinical manifestations, depending on which organ system is involved.

Cutaneous vasculitis is a histopathologic entity that is characterized by neutrophilic transmural inflammation of the vessel wall and is associated with fibrinoid necrosis, which is also termed leukocytoclastic vasculitis. Vasculitis has a wide spectrum of severity, ranging from skin-limited disease to life-threatening systemic involvement [1].

rG-CSF (filgrastim) is a myeloid growth factor produced by monocytes, macrophages, fibroblasts, and endothelial cells. The clinical use of rG-CSF includes recovery from neutropenia in patients receiving myelosuppressive chemotherapy for solid as well as hematologic malignancies; management of neutropenia deriving from other causes such as AIDS, genetic disorders in granulocyte production and, mobilization of peripheral blood progenitor cells [2, 3]. Some side effects have been reported for rG-CSF treatment: they are usually mild and they include bone pain, headache, and fatigue [3, 4].

Below, we report a case of leukocytoclastic vasculitis that occurred during rG-CSF treatment in a heart transplant patient.

## 2. Case Report

A 35-year-old man was admitted to our hospital because of leukopenia and anaemia. The patient had undergone heart transplantation (H-Tx) two months previously on account of dilated cardiomyopathy. After H-Tx, immunosuppression consisted in rabbit antithymocyte globulin (RATG) for three days (in doses that were regularly adjusted on the basis of absolute CD3+ T lymphocytes count), cyclosporine A, azathioprine, and steroids. No acute rejection episodes or infectious or autoimmune diseases were observed during followup.

At admission, the patient was on cyclosporin A (CyA: trough levels: 200 *μ*g/l), azathioprine (AZA: 100 mg/d), and steroids (5 mg/d). Laboratory data showed WBC = 1.5 × 10^3^/mcl, neutrophils = 0.9 × 10^3^/mcl, Hb = 8.0 g/dL, and platelets = 253 × 10^3^/mcl. Renal, hepatic, and blood coagulation proved to be within normal values. Autoimmunity, cryoglobulins, PCR values for EBV, CMV, HHV-8, and parvovirus B19 were negative. A peripheral blood smear showed severe leukopenia, anisopoikilocytosis, normal platelets morphology, and a lack of blast cells.

Bone marrow biopsy was performed. There was no evidence of bone marrow dysfunction such as myelodysplasia or infectious damage. The mature granulocytes count was found to be reduced.

AZA was stopped and rG-CSF therapy (filgrastim: 300 mcg s.c./d) was started to increase the patient's WBC count. Response was prompt: by day 3 of this therapy WBC had increased to 20 × 10^3^/mcl.

On day 4 of filgrastim, the patient developed tender, purple, circumscribed, and erythematous plaques on the lower legs; no fever was observed. Skin biopsy provided diagnosis of leukocytoclastic vasculitis. No serum antibodies were detected for nuclear cytoplasm antigens, hepatitis B and C virus, or HIV. Cryoglobulins, proteinuria, and p- and c-ANCA were negatives. Endomyocardial biopsy (EMB) was negative both for acute rejection and local vessel's vasculitis.

rG-CSF was discontinued and therapy with i.v. steroids was started. Complete resolution of vasculitis was achieved in two days. AZA was restarted, but after five days of treatment, WBC had again decreased to 1.5 × 10^3^/mcl, while absolute neutrophil count was 1.0 × 10^3^/mL after ten days. rG-CSF was restarted at a reduced dose, but, after one day, cutaneous vasculitis reappeared diffusely on legs and yet disappeared with the withdrawal of rG-CSF and systemic steroid therapy administration.

Patients received no further AZA, and haematological disorders were resolved in twenty days.

## 3. Discussion

Various cutaneous disorders have been associated with the use of rG-CSF. They include bullous pyoderma gangrenosum in subjects with preexisting eczema [5], Sweet's syndrome [6], and leukocytoclastic vasculitis [7].

From the clinical point of view, long-term data for rG-CSF therapy indicated some form of vasculitis as occurring in 4.1% of patients, at a rate that in patients with idiopathic neutropenia was slightly higher (5.9%) than it was in patients with congenital and cyclic types of neutropenia (resp., 3.3% and 3.1%) [8].

Various possible mechanisms have been proposed for cutaneous vasculitis. Shiohara et al. reported a substantial increase in IFN-y (a T-cell activator) in a patient with acetaminophen-induced vasculitis and disappearance of said activator upon discontinuation of the drug [9]. Elsner et al. proposed the involvement of altered Fcy receptor expression in the pathogenesis of vasculitis [10]. Zoellner et al. correlated rG-CSF activity with an enhancement of antibody-mediated toxicity by neutrophils, which are predominant in vasculitis lesions [11]. Finally, alterations in the surface markers and the chemotaxis of neutrophils have been conjectured by Spiekermann et al. [12].

A prompt examination of previous and current clinical data collected from our patient did not suggest he had taken any drugs that were known to induce vasculitis. Furthermore, no infection or autoimmune disease in the past or in current medical history was recorded.

With regard to the correlation between ANC and the development of cutaneous vasculitis, Cottle et al. reported that vasculitis generally increases and decreases in line, respectively, with increases and decreases in ANC values [13]. Moreover, most of the patients are able to continue rG-CSF, either at unchanged or at reduced doses [13].

In our patient, vasculitis appeared when the increase in WBC was high and responded well to systemic therapy, as previously reported. The relationship between the onset of vasculitis on the legs and rG-CSF was confirmed by the relapse of skin lesions when therapy was restarted again, albeit at a lower dosage.

Use of rG-CSF has been reported in kidney transplantation for treatment of leukopenia as induced by various drugs or by viral pathogens. Shorter leukopenic episodes were observed, along with significantly lower rate of infections. No side effects, including vasculitis, were reported [14].

In transplantation literature, it is still an open question whether rG-CSF therapy is harmful for organ grafts. Administration of rG-CSF not only increases neutrophils stimulation and differentiation but also leads to a limited rise in monocytes, the latter playing an important role in induction allograft rejection. This topic is controversial in the literature, which describes a very small number of such cases. Minguez et al. reported graft rejection in one patient on rG-CSF [15], whereas others did not [14, 16]. Furthermore, a recent paper from the United States Renal Transplantation Data System does not confirm a correlation between rG-CSF and the onset of acute rejection [17].

In our patient, an EMB was performed at the same time as the occurrence of vasculitis and proved the absence of any signs of rejection. Subsequent followup was invariably negative for acute rejection.
